# Schizophrenia Polygenic Risk and Experiences of Childhood Adversity: A Systematic Review and Meta-analysis

**DOI:** 10.1093/schbul/sbac049

**Published:** 2022-06-08

**Authors:** Grace E Woolway, Sophie E Smart, Amy J Lynham, Jennifer L Lloyd, Michael J Owen, Ian R Jones, James T R Walters, Sophie E Legge

**Affiliations:** MRC Centre for Neuropsychiatric Genetics and Genomics, Division of Psychological Medicine and Clinical Neurosciences, School of Medicine, Cardiff University, Cardiff, UK; MRC Centre for Neuropsychiatric Genetics and Genomics, Division of Psychological Medicine and Clinical Neurosciences, School of Medicine, Cardiff University, Cardiff, UK; MRC Centre for Neuropsychiatric Genetics and Genomics, Division of Psychological Medicine and Clinical Neurosciences, School of Medicine, Cardiff University, Cardiff, UK; MRC Centre for Neuropsychiatric Genetics and Genomics, Division of Psychological Medicine and Clinical Neurosciences, School of Medicine, Cardiff University, Cardiff, UK; MRC Centre for Neuropsychiatric Genetics and Genomics, Division of Psychological Medicine and Clinical Neurosciences, School of Medicine, Cardiff University, Cardiff, UK; MRC Centre for Neuropsychiatric Genetics and Genomics, Division of Psychological Medicine and Clinical Neurosciences, School of Medicine, Cardiff University, Cardiff, UK; MRC Centre for Neuropsychiatric Genetics and Genomics, Division of Psychological Medicine and Clinical Neurosciences, School of Medicine, Cardiff University, Cardiff, UK; MRC Centre for Neuropsychiatric Genetics and Genomics, Division of Psychological Medicine and Clinical Neurosciences, School of Medicine, Cardiff University, Cardiff, UK

**Keywords:** schizophrenia, polygenic risk score, child adversity, childhood trauma, gene- environment

## Abstract

**Background and Hypothesis:**

Schizophrenia has been robustly associated with multiple genetic and environmental risk factors. Childhood adversity is one of the most widely replicated environmental risk factors for schizophrenia, but it is unclear if schizophrenia genetic risk alleles contribute to this association.

**Study Design:**

In this systematic review and meta-analysis, we assessed the evidence for gene-environment correlation (genes influence likelihood of environmental exposure) between schizophrenia polygenic risk score (PRS) and reported childhood adversity. We also assessed the evidence for a gene-environment interaction (genes influence sensitivity to environmental exposure) in relation to the outcome of schizophrenia and/or psychosis. This study was registered on PROSPERO (CRD42020182812). Following PRISMA guidelines, a search for relevant literature was conducted using Cochrane, MEDLINE, PsycINFO, Web of Science, and Scopus databases until February 2022. All studies that examined the association between schizophrenia PRS and childhood adversity were included.

**Study Results:**

Seventeen of 650 identified studies met the inclusion criteria and were assessed against the Newcastle-Ottawa Scale for quality. The meta-analysis found evidence for gene-environment correlation between schizophrenia PRS and childhood adversity (*r* = .02; 95% CI = 0.01, 0.03; *P* = .001), but the effect was small and therefore likely to explain only a small proportion of the association between childhood adversity and psychosis. The 4 studies that investigated a gene-environment interaction between schizophrenia PRS and childhood adversity in increasing risk of psychosis reported inconsistent results.

**Conclusions:**

These findings suggest that a gene-environment correlation could explain a small proportion of the relationship between reported childhood adversity and psychosis.

## Introduction

Schizophrenia is a heritable condition with estimates of around 80% in twin and family studies.^1^ There have been recent major advances in our understanding of psychiatric genetic risk, driven by international collaborations, falling costs of genotyping technologies, and methodological advances.^2^ Genome-wide association studies (GWAS) have established that genetic risk for schizophrenia is polygenic, with a substantial contribution made by a large number of common genetic variants that individually increase risk to a small extent.^3,4^ Polygenic risk scores (PRS) are derived by calculating a weighted sum of genetic risk alleles present in an individual’s genome.^5^PRS is a measure of an individual’s inherited liability to developing a disorder or trait and, in schizophrenia, has accounted for increasing proportions of heritability as the power in GWAS datasets has increased, currently explaining up to 8% of the variance in liability.^6^

Environmental risk factors have also been shown to contribute to the likelihood of developing schizophrenia; evidence exists for illicit drug use (in particular cannabis),^7–10^ obstetric complications,^11–13^ season of birth,^14^ urbanicity,^15–17^ and migration.^18,19^ Childhood adversity is one of the most widely replicated environmental risk factors for psychosis,^20^ but the association has also been extended to schizophrenia.^21^ Childhood adversity is common worldwide and could involve anything that presents a serious threat to a child’s physical or psychological well-being such as trauma, abuse, neglect, parental death or separation, and bullying. A large meta-analysis across prospective cohorts, case-control, and cross-sectional design studies found that patients with psychosis were 2.78 (95% CI = 2.34, 3.31) times more likely to report being exposed to childhood adversity or trauma than controls.^22^ Furthermore, this association has demonstrated a “dose-effect” whereby as the severity of the childhood trauma increased, so did the likelihood of developing psychosis.^21^

Although evidence indicates that schizophrenia arises from both genetic liability and environmental exposures, how these risk factors combine to ultimately lead to disorder remains unclear. There is ongoing debate about whether genetic and environmental effects act independently, are associated via gene-environment *correlatio*n, and/or via gene-environment *interaction*. Table 1 provides a summary of these terms. A gene-environment correlation refers to how an individual’s genes can influence the environment they are exposed to. This can be either passive, evocative, or active. A passive gene-environment correlation refers to the association between the child’s genetics and the environment in which they are raised, whereby parents produce a home environment that is influenced by their genotypes.^23^ An evocative gene-environment correlation describes the association between a child’s genetically influenced behavior and the reaction from others in their environment to that behavior.^24^ Lastly, an active gene-environment correlation indicates an association between an individual’s genetically influenced traits and the selection of specific environments (such as a shy child seeking a different environment to an outgoing child). Twin and adoption studies have highlighted the importance of gene-environment correlations and interactions in human genetic research.^25^ The presence of a gene-environment correlation suggests that genetics may be a confounder that explains, at least in part, why an environmental risk factor is associated with a disorder or outcome. A gene-environment interaction alternatively suggests that an individual’s genetics can control (moderate) how sensitive they are to environmental exposures and whether those exposures will increase the risk of an outcome.^26,27^

**Table 1.** Key Terms

Term	Definition
Gene-environment correlation	An individual’s genotype or genetic liability is associated with the environment they are exposed to.
Passive gene-environment correlation	The parent’s genes affect the child-rearing environment, independent of child themselves.
Evocative gene-environment correlation	An individual’s genetically influenced traits evoke environmental responses from others.
Active gene-environment correlation	An individual selects specific environments based on their genetic propensity.
Gene-environment interaction	An individual’s genotype moderates the sensitivity to whether an environmental exposures increases the risk of disease.

*Note*: The table describes the key terms used in this review; a gene-environment correlation (which is subcategorized into passive, evocative, and active correlations) and a gene-environment interaction

The first studies investigating the relationship between genetic risk and childhood adversities used twin and family designs and found significant within-pair differences, indicating that at least part of the association between childhood trauma and psychosis is likely to be causal and not driven by gene-environment correlation.^28–30^ A recent qualitative review also found that childhood adversity was associated with psychosis largely independent of genetic liability.^31^ However, this review only included studies with individuals experiencing psychosis and where the genetic factor was analyzed as a mediator, thus limiting the studies that were included.

The aim of the current study was to conduct a systematic review and meta-analysis of the existing literature from both population and clinical samples investigating the gene-environment correlation between schizophrenia polygenic risk and reported childhood adversity. To our knowledge, this is the first quantitative review of this evidence. Secondly, we aimed to summarize the evidence regarding gene-environment interaction between schizophrenia polygenic risk and childhood adversity for the outcome of psychosis or schizophrenia case/control status. Better understanding the relationship between schizophrenia risk alleles and childhood adversity may enhance our knowledge of the mechanisms underpinning the development of psychosis and schizophrenia.

## Methods

We define childhood adversities as events that present a serious threat to a child’s physical or psychological well-being,^32^ and include maltreatment, domestic violence, assault, physical, sexual, emotional, or psychological abuse, neglect, bullying, discrimination, violence, victimization, parental loss, and bereavement. Broad definitions were used to try and capture as many studies as possible.

This review was registered on the PROSPERO website (https://www.crd.york.ac.uk/prospero/display_record.php?ID=CRD42020182812) and the PRISMA guidelines for reporting systematic reviews were followed^33^ (see supplementary tables 3 and 4).

We searched Cochrane, MEDLINE, PsycINFO, Web of Science, and Scopus databases from January 2007, when the first psychiatric GWAS was conducted,^34^ until February 2022. The search included all published and unpublished work that investigated schizophrenia PRS and childhood adversity. The following terms were used on each database: ((child* OR early life OR adolescen* OR development*)AND(trauma* OR advers* OR maltreat* OR molest* OR abuse OR stress* OR discrimination OR physical abuse OR sexual abuse OR emotional abuse OR neglect OR psychological abuse OR assault OR (violence OR domestic violence) OR (bullied OR bullying) OR victim* OR victimization OR parental loss OR bereave OR parental death* OR adopt* OR in care)) AND ((schiz* OR psychosis) AND (polygenic risk score OR PRS OR polygenic risk OR polyrisk score OR genome wide association stud* OR GWAS OR genetic score OR risk profile score OR genetic risk score OR genomic risk score OR GRS OR polygenic hazard score)).

**Fig. 1.**
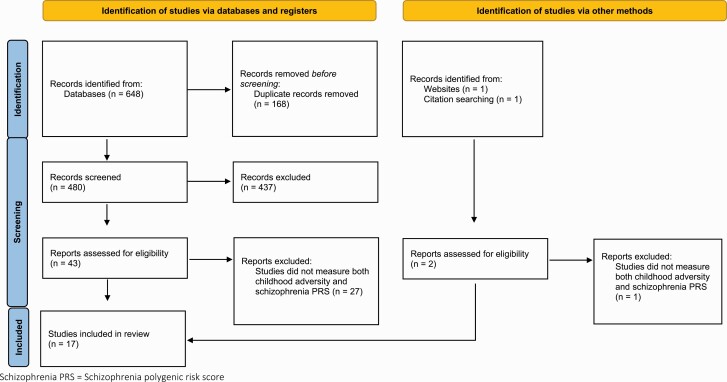
Study selection. The figure displays the study selection process for this review, arranged by studies identified via database searching and other methods as per 2020 PRISMA guidelines^35^.

All studies that measured an association between schizophrenia PRS and childhood adversity were included (see figure 1). Interaction studies were only included if psychosis or schizophrenia was the outcome of interest. Psychosis was broadly defined as any report of psychotic symptoms or psychotic disorders (including from self-report, assessment scales, and clinician diagnoses) and was included to capture studies of participants who did not meet diagnostic criteria for schizophrenia (eg, first-episode psychosis). Research was only included in human participants.

Initially, relevant titles and abstracts were identified independently by 2 researchers (G.E.W. and S.E.S.). Where the title and abstract were insufficient to determine eligibility, the full articles were obtained. The following data were extracted: author, publication type, study characteristics, number of participants, participant characteristics, method of recruitment, measurement of childhood adversity, effect size and standard error, power, and PRS *P*-value thresholds. Individual studies were assessed for quality using the Newcastle-Ottawa Scale for Quality of Observational Studies.^36^

We split the included studies into 2 groups based on the sample in which the research was conducted: (1) population studies and (2) clinical studies. We defined clinical studies as those that recruited patients with a diagnosis of schizophrenia or a related psychotic disorder (including children of those individuals) and population studies at those that have recruited participants from the general population.

### Meta-analysis

A multilevel meta-analysis of peer-reviewed studies investigating a gene-environment correlation was conducted using the *metafor*^37^ package in R. It was not possible to conduct a meta-analysis for gene-environment interaction due to the limited number of studies and different statistical models used. A 3-level meta-analysis was used to account for the nested structure in the data, whereby several childhood adversity outcomes were reported in the same study samples. Sample (as opposed to study) was used to define one level due to several studies being conducted in the same sample, for example, the Avon Longitudinal Study of Parents and Children (ALSPAC). Odds ratios and beta were first converted to standardized mean differences (Cohen’s *d*), and then to Pearson’s correlation coefficients using the *effectsize*^38^ R package to allow dichotomous and continuous data to be combined.^39,40^ If childhood adversity was analyzed as both a continuous and a binary variable, we selected the results from the continuous analysis to include the greatest amount of data.^38^ If studies reported multiple levels of adjustment, we extracted the most adjusted effect size (excluding other PRS scores).Where multiple PRS *P*-value thresholds were reported, we selected *P* <.05, given this threshold explains the most variance in schizophrenia case-control status.^3^ If this threshold was not available, or another PRS method was used, we selected the PRS that explained the most variance in childhood adversity. We excluded results from analyses involving PRS from multiple disorders or traits. Heterogeneity was assessed per level of the meta-analysis and meta-regression was used to explore possible causes of heterogeneity. Sensitivity analyses were also conducted to assess the effect of adding moderators into the analysis. Publication bias was assessed using funnel plots. Meta-analysis results are presented in a forest plot by population in which the effect size was reported.

## Results

A total of 650 studies were returned using the search criteria, 648 through database searches and 2 from additional sources. After removing duplicate studies, 480 studies were screened for relevance and 437 records were deemed unsuitable for this review and therefore excluded. Of the 45 full-text articles appraised, 17 studies met inclusion criteria and were evaluated as part of this review. Figure 1 illustrates the study selection and tables 2 and 3 contain a summary of each study included. All 17 studies were assessed for quality against the Newcastle-Ottawa Scale^36^ (supplementary tables 1 and 2), which was adapted to best capture the quality of the studies specific to this review; for example, studies did not score a point if the data were collected at only one timepoint during childhood (0–18). Although none of the studies scored less than 5/9, which would indicate a high risk of bias,^41^ 3 of the studies scored 5 which indicates potential bias.

**Table 2.** Summary of Population Studies

Study	Sample; Number of Participants	Childhood Adversity Investigated	Gene-Environment Correlation Between SZ PRS and CA	SZ PRS GWAS	Gene-Environment Interaction Between SZ PRS, CA, and Psychosis
Docherty et al (2018)^45^	S4S; 5947	Interpersonal trauma	*q*-value < 0.01^a^; effect size not reported^b^	PGC2 (2014)	Not assessed
Krapohl et al (2017)^53^	TEDS; 6710	Parental smacking	**β = .010, SE = 0.013, *X*** ^**2**^ **= 0.55, *P* = 5E-01**	PGC2 (2014)	Not assessed
Lehto et al (2020)^55^	UKBB; 3151 adopted	Adoption	**OR = 1.05; 95% CI = 1.01, 1.09; *P* = .01** ^a^	PGC2 (2014)	Not assessed
Leppert et al (2020)^52^	UKBB; 334 976 (total)	Sexually molested, felt loved, physically abused by family, felt hated by family, and someone to take to doctor	**Sexually molested: β = .062; 95% CI = 0.040, 0.084; *P* = 3.11E-08** ^a^ **Felt loved: β = −.044; 95% CI = −0.055, −0.032; *P* = 3.52E-14**^a^ **Physically abused by family: β = .033; 95% CI = 0.017, 0.048; *P* = 3.91E-05** **Felt hated by family: β = .060; 95% CI = 0.043, 0.077; *P* = 3.24E-12**^a^ **Someone to take to doctor: β = −.042; 95% CI = −0.058, −0.025; *P* = 6.36E-07**^a^	PGC2 (2014)	Not assessed
Pergola et al (2019)^24^	TRAILS; 650	Bullying victimization	Time point 1: *N* **=** 650, all *P* > .05^c^ Time point 2: *N* **=** 625, *F*_2,611_ **=** 3.4, *P* **=** .033, partial η ^2^ **=** 0.011 (ANOVA)^a^^,^^c^	PGC2 (2014)	Not assessed
Pries et al (2020)^46^	TwinssCan; 593	General adversity	**β = −.01; 95% CI = −0.03, 0.02; *P* = .676**	PGC2 + CLOZUK (2018)	Not assessed
Riglin et al (2019)^49^	ALSPAC; 8365	Peer victimization	**OR = 0.95; 95% CI = 0.86, 1.04; *P* = .292**	PGC2 (2014)	Not assessed
Sallis et al (2020)^42^	ALSPAC; 7426 (child PRS) MoBa; 7244 (child PRS)	Bullying, domestic violence, sexual abuse, emotional neglect, emotional cruelty, and physical cruelty	**ALSPAC any adversity across childhood and adolescence: OR = 1.14; 95% CI = 1.08, 1.20; *P* = 8.4E-6** Bullying: OR **=** 1.03; 95% CI **=** 0.97, 1.09; *P* **=** .342 Domestic violence: OR **=** 1.07; 95% CI **=** 1.01, 1.13; *P* **=** .028^a^ Sexual abuse: OR **=** 1.15; 95% CI **=** 1.03, 1.29; *P* **=** .012^a^ Emotional neglect: OR **=** 1.06; 95% CI **=** 0.96, 1.17; *P* **=** .235 Emotional cruelty: OR **=** 1.16; 95% CI **=** 1.09, 1.24; *P* **=** 3.49E-6^a^ Physical cruelty: OR **=** 1.12; 95% CI **=** 1.05, 1.20; *P* **=** 7.9E-4^a^ **MoBa: OR = 1.08; 95% CI = 1.02, 1.14; *P* = .005**	PGC2 (2014)	Not assessed
Schoeler et al (2019)^50^	ALSPAC; 5028	Bullying	**β = .038; 95% CI = 0.008, 0.068; *P* = .02** ^a^	PGC2 + CLOZUK (2018)	Not assessed
Ratanatharathorn et al (2021)^54^	NHS2; 13 313	Physical/emotional abuse, physical assault, and sexual abuse	**Physical/emotional abuse: OR = 1.11; 95% CI = 1.07, 1.16; *P* = 2.10E-08** ^a^ **Physical assault: OR = 1.09; 95% CI = 1.04, 1.13, *P* = 2.50E-05**^a^ **Sexual abuse: OR = 1.02, 95% CI = 0.98, 1.07, *P* = 2.80E-01**	PGC2 (2014)	Not assessed
Bolhuis et al (2021)^44^	Generation R; 1901	General adversity	***P***_***t***_ **< .5: OR = 1.08; 95% CI = 1.02,1 .15; *P* = .01 (Generation R)**^a^ ***P***_***t***_ **< .5: OR = 1.02; 95% CI = 1.01, 1.03; *P* = .001 (ALSPAC)**^a^	PGC2 (2014)	Not assessed
Peel et al (2021)^43^	TEDS; 3963	Emotional and physical abuse	**β = .44; 95% CI = 0.13, 0.75; *P* = .01** ^a^	PGC2 + CLOZUK (2018)	Not assessed
Results from controls in clinical population studies					
Trotta et al (2016)^48^	GAP; 110 unaffected community controls	General adversity Physical/sexual abuse, parental separation, parental death, taken into the care system, and number of family arrangements	**Controls: OR = 2.77; 95% CI = −1.92, 7.47; *P* = .247**	PGC2 (2014)	Assessed in cases
Aas et al (2021)^47^	EU-GEI; 690 controls	General adversity	Control: β **=** .03; 95% CI = −0.09, 0.25; *P* **=** .34 (binary) **Control: β = .09; 95% CI = 0.02, 0.16; *P* = .02**^a^ **(continuous)**	PGC2 (2014)	Assessed in cases
Guloksuz et al (2019)^58^	EU-GEI; 1542 controls	Bullying, emotional neglect, physical neglect, emotional abuse, physical abuse, and sexual abuse	**Bullying: OR = 1.28; 95% CI = 0.87, 1.89; *P* = .21** **Emotional abuse: OR = 1.13; 95% CI = 0.81, 1.59; *P* = .476** **Physical abuse: OR = 1.84; 95% CI = 1.19, 2.84; *P* = .006**^a^ **Sexual abuse: OR = 0.79; 95% CI = 0.51, 1.23; *P* = .292** **Emotional neglect: OR = 1.16; 95% CI = 0.90, 1.50; *P* = .258** **Physical neglect: OR = 1.19; 95% CI = 0.90, 1.58; *P* = .219**	PGC2 (2014)	Assessed in cases

*Note*: ALSPAC, Avon Longitudinal Study of Parents and Children; CA, childhood adversity; EU-GEI, EUropean Network of National Schizophrenia Networks Studying Gene-Environment Interactions; GAP, genes and psychosis; GWAS, genome-wide association studies; MoBa, Norwegian Mother, Father and Child Cohort Study; NHS2, Nurses’ Health Study 2; PGC2, Psychiatric Genomics Consortium 2^4^; PGC + CLOZUK, Psychiatric Genomics Consortium and CLOZUK^3^; SZ PRS, schizophrenia polygenic risk score; S4S, spit for science; TEDS, Twins Early Development Study; TRAILS, TRacking Adolescents’ Individual Lives Survey; TwinssCan, East Flanders Prospective Twin Survey; UKBB, UK BioBank. The table describes the population studies included in this review and outlines the number of participants with genetic data, the type of childhood adversity investigated, and the outcome.

^a^Study defined statistical significance. Effect sizes reported in bold were used in the meta-analysis.

^b^Study not included in meta-analysis due to no effect size.

^c^Study not included in meta-analysis because not peer reviewed.

**Table 3.** Summary of Clinical Studies

Study	Sample; Number of Participants	Childhood Adversity Investigated	Gene-Environment Correlation Between SZ PRS and CA	SZ PRS GWAS	Gene-Environment Interaction Between SZ PRS, CA, and Psychosis
Guloksuz et al (2019)^58^	EU-GEI; 1699 schizophrenia spectrum patients and 1542 controls	Bullying, emotional neglect, physical neglect, emotional abuse, physical abuse, and sexual abuse	Not assessed	PGC2 (2014)	Positive interaction between SZ PRS, case status, and bullying (RERI **=** 2.76; 95% CI **=** 0.29, 5.23; *P* **=** .028^a^), emotional abuse (RERI **=** 5.52; 95% CI **=** 2.29, 8.75; *P* < .001^a^), emotional neglect (RERI **=** 2.46; 95% CI **=** 0.98, 3.94; *P* **=** .001^a^), and sexual abuse (RERI **=** 7.61; 95% CI **=** 2.05, 13.17; *P* **=** .007^a^). No interaction found for physical abuse (RERI **=** 1.64; 95% CI **=** −1.07, 4.34; *P* **=** .235) and physical neglect (RERI **=** 1.51; 95% CI **=** 0.00, 3.03; *P* **=** .051).
Trotta et al (2016)^48^	GAP; 80 first-episode psychosis cases and 110 unaffected community controls	Physical/sexual abuse, parental separation, parental death, taken into the care system, and number of family arrangements	**Cases: OR = 1.71; 95% CI = −5.37, 8.78; *P* = .636**	PGC2 (2014)	No significant interaction (*b* **=** −0.20; SE **=** 0.41; *P* **=** .632)
Zwicker et al (2020)^57^	FORBOW; 297 children of parents with psychiatric diagnoses	Childhood maltreatment and peer victimization	**Overall adversity: β = .02; 95% CI = −0.15, 0.18; *P* = .861** **Socioeconomic adversity: β = −.05; 95% CI = −0.20, 0.10; *P* = .502** **Victimization: β = .05; 95% CI = −0.13, 0.23; *P* = .564**	PGC2 (2014)	Not assessed
Lemvigh et al (2021)^59^	Vulnerability Indicators of Psychosis study; 56 proband pairs (schizophrenia spectrum), 49 healthy control pairs, 6 probands without siblings (total *n* **=** 216)	General adversity	Not assessed	PGC2 (2014)	No significant interaction: OR **=** 1.00, 95% CI: 0.48, 2.07, *P* **=** .999
Aas et al (2021)^47^	EU-GEI; 384 first-episode psychosis cases and 690 controls	General adversity	Case: β **=** .02; 95% CI = −0.14, 0.22; *P* **=** .65 (binary) **Case: β = .02; 95% CI = −0.08, 0.11, *P* = .74 (continuous)**	PGC2 (2014)	Positive interaction between SZ PRS and CA: ICR **=** 1.28, 95% CI = −1.29, 3.85

*Note*: Abbreviations are explained in the first footnote to table 2. FORBOW, Families Overcoming Risks and Building Opportunities for Well-being; ICR, interaction contrast ratio; RERI, relative excess risk due to interaction. The table describes the clinical studies included in this review and outlines the number of participants with genetic data (by case/control status), the type of childhood adversity investigated, and the outcome.

Effect sizes reported in bold were used in the meta-analysis.

^a^Study defined statistical significance.

In this review, we discuss the evidence for a gene-environment correlation between reported childhood adversity and schizophrenia PRS, separately by population and clinical studies, and present the findings from a meta-analysis of this evidence. We then outline the evidence for a gene-environment interaction in the context of psychosis or schizophrenia.

### Gene-Environment Correlation

Of the 16 studies that investigated a gene-environment correlation between childhood adversity and schizophrenia PRS, 9 of the 12 population-based studies showed evidence for association (across multiple adversities), but effect sizes found were small. Studies in clinical samples reported inconsistent findings and were hampered by small sample size.

### Population Studies

There was variability in the way childhood abuse was defined in the included studies. Childhood adversity was considered as a broad definition (ie, any adversity) either in binary form or on a continuous scale, or as more specific forms such as bullying or abuse (table 2). First, we consider studies that defined childhood adversity as a broad construct.

In one of the largest studies conducted, Sallis et al assessed any adversity across childhood and adolescence and found an association with schizophrenia PRS in both the Avon Longitudinal Study of Parents and Children longitudinal cohort (ALSPAC; OR = 1.14;95% CI = 1.08, 1.20; *P* = 8.4E-6) and in the Norwegian Mother, Father and Child Cohort Study (MoBa; OR = 1.08; 95% CI = 1.02, 1.14; *P* = .005).^42^ A study in a sample of 3963 unrelated twins (Twins Early Development Study) found retrospectively self-reported childhood trauma to be associated with higher schizophrenia PRS (β = .44; 95% CI = 0.13, 0.75; *P* = .01).^43^ Furthermore, a prospective birth cohort (Generation R) of 1901 participants found schizophrenia PRS to be associated with maternal reports of childhood adversity (OR = 1.08; 95% CI = 1.02, 1.15; *P* = .01) which the authors replicated in the ALSPAC cohort (OR = 1.02; 95% CI = 1.01, 1.03; *P* = .001).^44^ Further analyses demonstrated that this association was driven by childhood adversities occurring before the age of 5 years (OR = 1.20; 95% CI = 1.05, 1.36; *P* < .01). A study of 5947 participants from a university student population (age 18–22) found that schizophrenia PRS predicted a report of previous trauma, although the study is limited by its retrospective nature and the lack of information on the timing of the trauma.^45^

No association was found in 593 twins, where schizophrenia PRS and childhood adversity were investigated to understand the influence on momentary mental state domains (including subtle psychosis expression and emotional affect; β = −.01; 95% CI = −0.03, 0.02; *P* = .676).^46^ Finally, 2 clinical studies^47,48^ assessed the relationship between global childhood adversity and schizophrenia PRS in their control samples and both found positive associations, although only one was statistically significant.^44^ These studies are discussed further in the “Clinical Studies” section.

Bullying and peer victimization are the most researched specific forms of childhood adversity in population-based research studies. A large study in 8365 participants from ALSPAC found that schizophrenia PRS was not associated with exposure to child victimization (OR = 0.95; 95% CI = 0.86, 1.04; *P* = .292).^49^ A subsequent follow-up study in this sample including data from age 13, however, reported a positive association with schizophrenia PRS (β = .038; 95% CI = 0.008, 0.068; *P* = .02).^50^ A further study in ALSPAC, using a range of questionnaire responses collected throughout childhood and adolescence, reported no association between bullying and schizophrenia PRS; however, evidence was found for an association in MoBa (OR = 1.09; 95% CI = 1.03, 1.15; *P* = .003) which assessed sibling violence (occurring in the home environment),^42^ suggesting there could be qualitative differences between sibling and peer bullying. The final study investigating bullying victimization^24^ in 650 participants through peer nomination scores reported a significant association with schizophrenia PRS at 14 years of age.^24^ In addition, the researchers found bullying victimization mediated the relationship between schizophrenia PRS and later psychotic experiences.^24^ The variability in definitions of adverse peer experiences across these studies is also likely to have contributed to the inconsistent findings reported. Bullying and peer victimization have been shown to be qualitatively different experiences, with bullying being more severe.^51^

There are 3 studies that have explored other specific childhood adversities. Trauma questionnaire responses were examined in 334 976 participants in the UK Biobank cohort.^52^ They tested 5 phenotypes related to childhood adversity and found 4 to be significantly associated; participants with higher schizophrenia PRS were more likely to report sexual abuse as a child (β = .062; 95% CI = 0.040, 0.084; *P* = 3.11E-08), feeling hated by a family member as a child (β = .060; 95% CI = 0.043, 0.077; *P* = 3.24E-12), and were less likely to report feeling loved as a child (β = −.044; 95% CI = −0.055, −0.032; *P* = 3.52E-14) or that they had someone to take them to the doctors as a child (β = −.042; 95% CI = −0.058, −0.025; *P* = 6.36E-07).^52^ The second study used a twin cohort of 6710 participants to explore parental smacking during childhood and found no evidence of an association between schizophrenia PRS and parent smacks or slaps as a child (β = .010, SE = 0.013, *X*^2^ = 0.55, *P* = .50).^53^ Thirdly, a recent study of a cohort of 13 313 female nurses found schizophrenia PRS was associated with a higher risk of experiencing physical or emotional abuse (OR = 1.11; 95% CI = 1.07, 1.16; *P* = 2.10E-08) and physical assault (OR = 1.09; 95% CI = 1.04, 1.13; *P* = 2.50E-05) but not sexual abuse (OR = 1.02; 95% CI = 0.98, 1.07; *P* = .28).^54^

All of the studies discussed so far in the review have assessed the gene-environment correlation between schizophrenia PRS of the child and childhood adversity, but their experimental designs did not permit further dissection into the specific types of gene-environment correlation (ie, passive, evocative, active, described in table 1). Two studies have further explored the association between schizophrenia PRS and childhood adversity by also studying the parents’ PRS or using an adoption study design.

Sallis et al (described above) also assessed the association of childhood trauma with the schizophrenia PRS in the child’s parents in the ALSPAC and MoBa samples^42^ and found positive associations in the mothers (OR = 1.13)^42^ and fathers showed a similar trend (OR = 1.04). It is argued by the authors that these findings provide evidence for a passive gene-environment correlation as the schizophrenia PRS in the mother was associated with her child’s adversity and thus the effects are unlikely to be driven solely by genetic effects from the child. Hypothetically, if an evocative or active gene-environment correlation was observed (ie, driven by the child’s genetic risk only), a stronger effect of the child’s PRS would have been found.^42^ A study in 3151 adopted individuals from the UK Biobank explored the association of schizophrenia PRS with childhood adoption.^55^ Childhood adoption is an indicator of early-life adversity and is associated with increased rates of mental health problems (including nonaffective psychoses such as schizophrenia) in later life.^56^ The researchers found that for each SD increase in schizophrenia PRS, the odds of being adopted increased by 5%. The authors argue these findings indicate support for a passive gene-environment correlation, whereby the child’s genetic predisposition is associated with the child’s environment, in this case, being adopted.

An evocative gene-environment correlation has been frequently used as a potential explanation for how bullying could theoretically mediate the genetic risk of later psychotic symptoms, by which a child with a high schizophrenia PRS may be more likely to evoke certain behaviors such as bullying from others.^24^ However, this mechanism has not been empirically demonstrated to date and distinguishing between different theoretical mechanisms of gene-environment correlations is challenging and requires PRS for the parents.

### Clinical Studies

Two studies investigated this question in clinical cohorts consisting of patients with a diagnosis on the schizophrenia spectrum or first-episode psychosis. A further study investigated children of parents with a schizophrenia spectrum, bipolar or major depressive disorder diagnosis.

In 384 individuals with first-episode psychosis, a recent study found an association between schizophrenia PRS and childhood adversity in 690 unaffected controls (β = .09; 95% CI = −0.02, 0.16; *P* = .02) but not in cases (β = .02; 95% CI = −0.08, 0.11; *P* = .74) when considering childhood adversity as a continuous variable.^47^ A further study of 80 first-episode psychosis patients and 110 controls found that while participants experiencing psychosis reported significantly more childhood adversity than controls, and patients had a higher schizophrenia PRS, the schizophrenia PRS was not significantly associated with childhood adversity in either patients (OR = 1.71; 95% CI = −5.37, 8.78; *P* = .636) or unaffected controls (OR = 2.77; 95% CI = −1.92, 7.47; *P* = .247).^48^ Lastly, a cohort of 297 children at clinical high risk due to having a parent with a diagnosis of major depressive disorder, bipolar disorder, or a schizophrenia spectrum disorder found no evidence of a significant relationship between schizophrenia PRS and peer victimization (β = .05; 95% CI = −0.13, 0.23; *P* = .564), overall adversity (β = .02; 95% CI = −0.15, 0.18; *P* = .861), or social-economic adversity (β = −.05; 95% CI = −0.20, 0.10; *P* = .502).^57^

### Meta-analysis

**Fig. 2.**
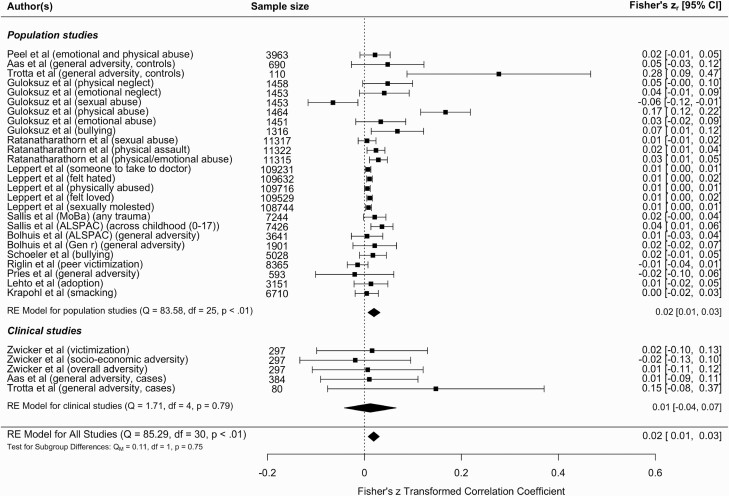
Meta-analysis of schizophrenia polygenic risk score and childhood adversity. The figure displays the meta-analysis of all the studies that reported a gene-environment correlation between childhood adversity and schizophrenia polygenic risk. The meta-analysis is subcategorized into population studies and clinical studies, with the effect for each population displayed at the end of the sections and the overall effect displayed at the bottom of the figure. Two of the studies (Aas et al^47^ and Trotta et al^48^) have results in both population and clinical subgroups to reflect the effect sizes reported separately in cases and controls. The Guloksuz et al^58^ study only appears in the population section, as a gene-environment correlation effect size was only reported in controls, not cases. If studies reported effect sizes in 2 samples, the sample is identified in brackets, as are the specific types of adversity.

We carried out a multilevel meta-analysis of all peer-reviewed studies investigating a gene-environment correlation between reported childhood adversity and schizophrenia PRS (figure 2) and found evidence for a significant correlation (*r* = .02; 95% CI = 0.01, 0.03; *P* = .001) (equivalent to OR = 1.07; 95% CI = 1.03, 1.12). The observed effect was statistically significant in population-based studies (*r* = .02; 95% CI = 0.01, 0.03; *P* = .002) (equivalent to OR = 1.07; 95% CI = 1.03, 1.12) but not clinical studies (*r* = .01; 95% CI = −0.04, 0.07; *P* = .658) (equivalent to OR = 1.04; 95% CI = 0.86, 1.27). In a multilevel meta-analysis, heterogeneity is reported per level; *I*^2^_level 3_ = 17.89% of the total variation was attributed to the between-cohort level, and *I*^2^_level 2_ = 69.73% was attributed to within-cohort heterogeneity. However, leave-one-out analyses demonstrated that one study^57^ was very influential and when removed from the analysis, the heterogeneity statistics demonstrated instead that just over 20% of the variance can be explained by between-cohort heterogeneity (*I*^2^_level 2_ = 0%; *I*^2^_level 3_ = 24.19%) (supplementary figure 1).

The funnel plot indicated that the meta-analysis was unlikely to be affected by significant publication bias (supplementary figure 2) but it highlighted 2 studies that could be considered as outliers.^47,57^ Removing these 2 studies from the meta-analysis reduced the correlation slightly but it remained significant (*r* = .012; 95% CI = 0.01, 0.02; *P* = 1.50E-5). Moderators of population versus clinical study (*F*_1_ = 0.11, *P* = .75) and effect size reported in original study (β/OR) (*F*_1_ = 2.14, *P* = .14) were not significant when included in the meta-analysis. Prediction intervals for the overall model ranged from *r* = −.0213 to .0595 (OR = 0.93, 1.24), and ranged from *r* = −.0237 to .0631 (OR = 0.92, 1.26) for population studies, and *r* = −.0415 to .0656 (OR = 0.86, 1.27) for clinical studies.

We conducted subanalyses of specific childhood adversities, which showed a significant association in studies reporting an overall adversity score (eg, total childhood trauma questionnaire score) (*r* = .024; 95% CI = 0.01, 0.04; *P* < .0001) but not for physical abuse (*r* = .063; 95% CI = −0.03, 0.16; *P* = .205), sexual abuse (*r* = −.010; 95% CI = −0.05, 0.03; *P* = .616), and peer victimization (*r* = .017; 95% CI = −0.02, 0.05; *P* = .370) when considered separately.

### Gene-Environment Interaction

Four clinical studies investigated the evidence for an interaction between schizophrenia PRS and childhood adversity in the context of psychosis or schizophrenia case/control status. The 2 larger studies reported evidence for a positive additive interaction, while the 2 smaller studies found no evidence of a gene-environment interaction.

In 1699 schizophrenia spectrum cases and 1542 controls, Guloksuz et al^58^. demonstrated an additive interaction of genetic liability for schizophrenia spectrum disorder on the risk of developing psychosis and bullying (relative excess risk due to interaction [RERI] = 2.76; 95% CI = 0.29, 5.23; *P* = .028), emotional abuse (RERI = 5.52; 95% CI = 2.29, 8.75; *P* < .001), emotional neglect (RERI = 2.46; 95% CI = 0.98, 3.94; *P* = .001), and sexual abuse (RERI = 7.61; 95% CI = 2.05, 13.17; *P* = .007). No additive interactive effect was evident between schizophrenia PRS and physical abuse or physical neglect. The authors argued that both emotional and sexual abuse showed a “mechanistic interaction,” meaning that some individuals would only develop schizophrenia if both genetic risk for schizophrenia and emotional or sexual abuse was present, but not one alone.

Aas et al^47^ used another method, the interaction contrast ratio (ICR) [OR_exposure_ and PRS − OR_exposure_ – OR PRS + 1], to assess an additive interaction. They reported evidence of a nonsignificant positive interaction between schizophrenia PRS and childhood adversity (ICR = 1.28, 95% CI = −1.29, 3.85). When analyzing the interaction by subtype, the strongest interactions found were physical abuse (ICR = 6.25, 95% CI = −6.25, 20.88) and physical neglect (ICR = 3.68, 95% CI = −1.69, 9.06). However, all the CIs were large and included zero.

In 80 first-episode psychosis cases and 110 unaffected controls, Trotta et al found no evidence of an additive interaction between schizophrenia PRS and childhood adversity (*b* = −0.20; SE = 0.41; *P* = .632).^48^ Lastly, a Danish twin study in 56 schizophrenia spectrum proband pairs, 49 healthy control pairs, and 6 individual probands found no evidence of an interaction between schizophrenia PRS and childhood adversity (OR = 1.00, 95% CI = 0.48, 2.07, *P* = .999).^59^

The inconsistent findings relating to gene-environment interaction are likely driven by small sample sizes and differing statistical approaches in defining interaction.

## Discussion

In this systematic review and meta-analysis, we investigated the evidence for a gene-environment correlation or interaction between schizophrenia PRS and reported childhood adversity to increase vulnerability to psychosis. A total of 26 gene-environment associations were reported from 15 population-based studies. Of these, 18 showed evidence for an association at *P* < .05 between schizophrenia PRS and childhood adversity, but effect sizes reported were small. Results from the meta-analysis, which controlled for the nonindependence of these associations due to overlapping samples, complimented these conclusions by demonstrating a significant gene-environment correlation between schizophrenia PRS and childhood adversity (*r* = .02; 95% CI = 0.01, 0.03; *P* = .001). Only 4 studies investigated a gene-environment interaction between schizophrenia PRS and self-reported childhood adversity on psychosis risk and reported inconsistent findings.

Based on the current literature and results from this meta-analysis, there is evidence to support a gene-environment correlation between schizophrenia PRS and childhood adversity in the general population. The population-based studies providing support for a gene-environment correlation included evidence from high-quality, prospective, and large cohort studies including UK Biobank, ALSPAC, MoBa, Generation R, Nurses’ Health Study 2 (NHS2), and Twins Early Development Study (TEDS).^42–44,50,54,55^ The effect sizes relating to schizophrenia PRS detected in these samples were small, and the overall correlation detected in our meta-analysis was *r* = .02 (95% CI = 0.01, 0.03; corresponding to an OR of 1.07 [95% CI = 1.03, 1.12] for any adversity). The small amount of variance explained demonstrates that schizophrenia PRS is making a limited contribution to the occurrence of childhood adversity. This study indicates that a gene-environment correlation is unlikely to fully explain the relationship between childhood adversity and increased risk of experiencing psychosis, although we have only assessed the contribution of common genetic risk captured by PRS and have been unable to statistically quantify the exact amount of this association that is explained by schizophrenia PRS. To definitively quantify this gene-environment correlation, consistent definitions of schizophrenia and psychosis need to be used, and the contribution of rare variation also needs to be quantified. Moreover, PRS from currently powered GWAS only capture a small proportion (around 8%) of the variance in liability for schizophrenia^6^; hence, any PRS estimate would provide a lower bound estimate, and more accurate estimates may be found using twin and family studies. Nevertheless, our findings are supported by earlier twin and family studies that reported significant within-pair differences,^28–30^ and thus suggested that at least part of the association between childhood trauma and psychosis is likely to be causal, or at least not driven by gene-environment correlation. Schizophrenia PRS has been associated with other mental health problems in adolescence aside from psychosis such as anxiety,^60^ indicating that the gene-environment correlation found in this study may have implications beyond the pathway to psychosis.

There were only a few studies, with limited sample sizes, investigating evidence of a gene-environment correlation in individuals with schizophrenia, related psychotic disorders, or psychotic symptoms. Although the findings were not statistically significant, clinical studies seemed to follow a similar pattern of effect to the population-based studies, which was mirrored in the meta-analysis findings. Studies investigating this relationship in clinical studies could be at risk of collider bias as schizophrenia PRS and childhood adversity are both associated with psychosis. However, as the meta-analysis demonstrates the correlation in population studies, it seems unlikely to be impacting the results. The lack of evidence for a gene-environment correlation in the clinical samples is likely due to insufficient power to detect an effect but it is possible the association in clinical and population samples are not consistent.

We found inconsistent findings in the few studies that investigated gene-environment interaction between schizophrenia PRS and childhood adversity. The largest clinical sample showed an additive interaction effect between schizophrenia PRS and childhood adversity in relation to psychosis risk.^58^ These findings are further supported by previous studies in twin, adoption, and family studies in schizophrenia.^26^ No statistically significant evidence was found in a subpopulation of first-episode psychosis cases in the same sample^47^ as all the CIs included zero. The 2 other studies^48,59^ did not find evidence of an additive interaction but it is likely the studies were underpowered. It is possible that other studies ran interaction analyses but did not report negative results, resulting in publication bias. However, different interaction models were used across these studies which has been demonstrated to impact consistency of findings.^61^ Additive and multiplicative models of interactions have different null hypotheses and interactions can be identified using one method and not using the other in certain circumstances.^62^ The variance in our findings are by no means unique, gene-environment interactions of complex diseases are challenging to research and difficulties are seen across the field.^63^ Furthermore, even when significant interactions have been observed, it has been argued that their contribution to advancing knowledge of disease is minimal.^61^

We found considerable inconsistencies across the literature and one of the main differences was the diverse range of childhood adversity measures used and the way in which they were scored. This may be particularly important as we found heterogeneous strengths of association based on adversity type in our meta-analysis. Even within specific adversities differences prevailed, for example, for peer victimization and bullying, a combination of peer nomination scores, teacher reports, parent responses, and child’s reports were obtained, making differences hard to interpret. We found that the measure of childhood adversity was at most risk of bias when scoring the Newcastle-Ottawa Quality Assessment Scale,^36^ primarily due to the use of retrospective self-reports. Given the sensitivity of childhood adversity, alternative solutions to a self-report method of data collection are at risk of other biases and so addressing this risk of bias is not simple. Retrospective reports could lead to error as people misremember over time (misclassification bias). This could be nondifferential misclassification, which could affect people equally in relation to their PRS status and almost always leads to an underestimation of an association, or differential misclassification which could affect people differently depending on their PRS. For example, those with high PRS are more likely to get schizophrenia and to develop cognitive deficits that could affect their memory,^64^ which could lead to either an under or overestimate of the association. Moreover, individuals who have a psychiatric diagnosis may be more likely to report childhood adversity by means of an explanation as to why they became unwell, resulting in confirmation bias. Conversely, high-quality longitudinal studies such as ALSPAC may have other forms of bias as a result of participant attrition, of relevance to this study is the association with schizophrenia PRS, that could have influenced results.^65^ Lastly, most of the studies included in our review measured the quantity of childhood trauma, rather than disentangling the other complexities that are involved within genetic-environment interactions such as timing, duration, severity, and extent of repeated exposures.^66,67^

A considerable limitation of the literature is that all studies have been conducted in individuals of European ancestries. Currently, schizophrenia PRS is not as predictive of schizophrenia case-control status in non-European ancestries as the majority of the genetic studies are in individuals of European ancestries.^6^ Therefore, it is unknown whether these findings are generalizable to individuals from different populations. This is especially important given the increased risk of schizophrenia in ethnic minority populations.^68^ In addition, PRS were also derived differently across the studies. There were differences among the schizophrenia GWAS summary statistics (PGC2/PGC2 + CLOZUK^3,4^), genotyping arrays, standardization reference group, and method. Furthermore, it is likely that many of these studies did not have adequate power to detect what is likely to be a small effect.

### Recommendations

Future research should include those from non-European ancestries, be conducted in larger clinical samples, incorporate rare genetic variation, and could consider including measures of childhood adversity from anonymized online data or using data linkage databanks. Researchers should also include measures of age, duration, type of adversity, and severity when investigating childhood adversity to explore other dimensions of the relationship between schizophrenia PRS and childhood adversity. Studies should also consider the inclusion of parental PRS to enable researchers to disentangle the nature of gene-environment correlation. Multiple PRS thresholds and power analyses should be used in future studies to demonstrate robust methodologies^69^ and more contemporary PRS methods such as PRS-CS^70^ should be considered.

## Conclusion

We have reviewed all studies investigating a gene-environment correlation and gene-environment interaction between schizophrenia PRS and reported childhood adversity. A multilevel meta-analysis demonstrates a small, yet significant gene-environment correlation between schizophrenia PRS and childhood adversity, indicating that a gene-environment correlation is likely to explain a minimal part of the association between childhood trauma and psychosis. The small effect size estimate is, however, dependant on the power of currently available PRS and more variance may be explained using other study designs and as GWAS become more powered. Due to the limited number of studies investigating gene-environment interaction, we were unable to conclude whether childhood adversity interacts with genetic predisposition to schizophrenia to increase the risk of psychosis. The methodological differences and limitations emphasize the need for further research, especially in clinical cohorts.

## Supplementary Material

sbac049_suppl_Supplementary_Material
