# Correction to: Schizophrenia Polygenic Risk and Experiences of Childhood Adversity: A Systematic Review and Meta-analysis

**DOI:** 10.1093/schbul/sbad006

**Published:** 2023-03-16

This is a correction to: Grace E Woolway, Sophie E Smart, Amy J Lynham, Jennifer L Lloyd, Michael J Owen, Ian R Jones, James T R Walters, Sophie E Legge, Schizophrenia Polygenic Risk and Experiences of Childhood Adversity: A Systematic Review and Meta-analysis, *Schizophrenia Bulletin*, Volume 48, Issue 5, September 2022, Pages 967–980, https://doi.org/10.1093/schbul/sbac049

Referencing errors were identified in the original published version of this manuscript. These errors have now been corrected.

